# 

**Published:** 1876-04

**Authors:** 


### *BOOK NOTICE.*

THE MESSRS. PETERSON, of Philadelphia, sent us an elegant volume, "The Legends of the American Revolution," by George Lippard. It is devoted to the records and legends of the American Revolution, which it embodies in a series of original and historical pictures, the result of five years' labor on the part of the author, and comprises his researches into the archives, documents and papers hidden away in libraries and closets. It also combines those traditions which old men have brought down from their parents to our time, concerning the days of "1776." It is a series of battle pictures, where the heroes are made living, present and visible to our senses. Here we do not merely turn over the dead dry facts of General Washington's battles, as if coldly digging them out of their tomb—but we see the living General as he moves around over the field of glory. It comprises life-like descriptions of the battles of Germantown, Saratoga, Quebec, Brandywine, Trenton, Paoli, Red Bank, etc., with a new and minute description of the Signing and Proclamation of the "Declaration of Independence." The work is entertaining to all readers but possesses especial interest to boys and girls, who are captivated by its vivid descriptions, and accordingly greedily absorb the historical information it contains. The volume contains a fine steel engraving of the Battle of Germantown. The price is $1.50 in paper cover, and $2 in morocco cloth. It will be sent by Messrs. Peterson, free by mail, on receipt of price.

As Summer approaches, all prudent parents of young children should provide themselves with at least one package of little sugar pellets, known as "Van Deren's Remedy." It is warranted to be an *infallible* remedy for all bowel complaints of children. Price 50 cents. Sent free on recept of price, by Hall & Ruckel, Wholesale Druggists, 218 and 220 Greenwich Street, New York.

